# Supplementary material for: Causal interplay between lactose intolerance and gut microbiota: a combined bidirectional Mendelian randomization and in vivo validation study
Source: Front Nutr. 2026 Jun 1;13:1803337. doi: 10.3389/fnut.2026.1803337 (PMC13265576; doi:10.3389/fnut.2026.1803337)
Supplement: Supplementary file 1 [file Data_Sheet_1.zip › supplementary materials/Forward/leave one out cross validation/ebi-a-GCST90027854.finngen_R12_E4_LACTONAS.pdf]

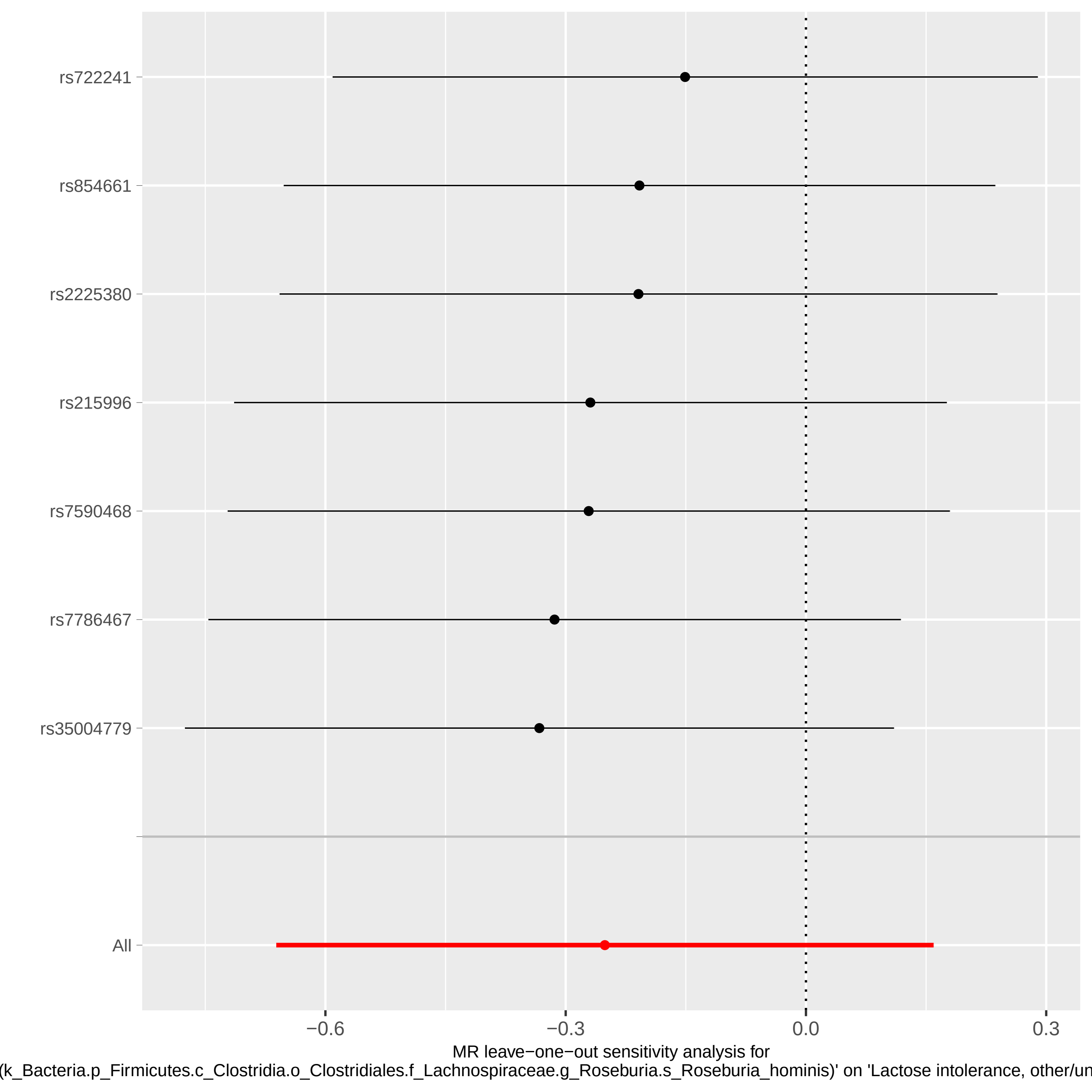

rs722241
rs854661
rs2225380
rs215996
rs7590468
rs7786467
rs35004779
All
−0.6
−0.3
0.0
0.3
MR leave−one−out sensitivity analysis for
(k_Bacteria.p_Firmicutes.c_Clostridia.o_Clostridiales.f_Lachnospiraceae.g_Roseburia.s_Roseburia_hominis)' on 'Lactose intolerance, other/un
